# Mesenchymal Stem Cells Shift Mitochondrial Dynamics and Enhance Oxidative Phosphorylation in Recipient Cells

**DOI:** 10.3389/fphys.2018.01572

**Published:** 2018-11-13

**Authors:** Christopher Newell, Rasha Sabouny, Dustin. S. Hittel, Timothy E. Shutt, Aneal Khan, Matthias S. Klein, Jane Shearer

**Affiliations:** ^1^Department of Medical Genetics, Cumming School of Medicine, University of Calgary, Calgary, AB, Canada; ^2^Department of Biochemistry & Molecular Biology, Cumming School of Medicine, University of Calgary, Calgary, AB, Canada; ^3^Departments of Pediatrics, Cumming School of Medicine, University of Calgary, Calgary, AB, Canada; ^4^Department of Food Science and Technology, The Ohio State University, Columbus, OH, United States; ^5^Faculty of Kinesiology, University of Calgary, Calgary, AB, Canada

**Keywords:** metabolism, high-fat diet, metabolic inflammation, hepatic, mitochondrial regulation

## Abstract

Mesenchymal stem cells (MSCs) are the most commonly used cells in tissue engineering and regenerative medicine. MSCs can promote host tissue repair through several different mechanisms including donor cell engraftment, release of cell signaling factors, and the transfer of healthy organelles to the host. In the present study, we examine the specific impacts of MSCs on mitochondrial morphology and function in host tissues. Employing *in vitro* cell culture of inherited mitochondrial disease and an *in vivo* animal experimental model of low-grade inflammation (high fat feeding), we show human-derived MSCs to alter mitochondrial function. MSC co-culture with skin fibroblasts from mitochondrial disease patients rescued aberrant mitochondrial morphology from a fission state to a more fused appearance indicating an effect of MSC co-culture on host cell mitochondrial network formation. *In vivo* experiments confirmed mitochondrial abundance and mitochondrial oxygen consumption rates were elevated in host tissues following MSC treatment. Furthermore, microarray profiling identified 226 genes with differential expression in the liver of animals treated with MSC, with cellular signaling, and actin cytoskeleton regulation as key upregulated processes. Collectively, our data indicate that MSC therapy rescues impaired mitochondrial morphology, enhances host metabolic capacity, and induces widespread host gene shifting. These results highlight the potential of MSCs to modulate mitochondria in both inherited and pathological disease states.

## Introduction

Initially identified as plastic-adherent cells, mesenchymal stem cells (MSCs) are non-hematopoietic stem cells that are capable of differentiating into a multitude of cell lineages (Colter et al., 2000; Maijenburg et al., 2012). Presenting great promise for regenerative medicine and gene-based therapy, MSCs are further characterized by their capacity for self-renewal and the ability to expand as isolated cells *in vitro* (Hsu et al., 2016). Known to preferentially home to sites of injury and inflammation, administered MSCs have exhibited promotion of host tissue repair through donor cell engraftment, release of cell signaling factors, and transfer of healthy organelles including mitochondria (Shi et al., 2010; Bernardo and Fibbe, 2012; Islam et al., 2012).

At present, little is known about the impact of MSC therapy on mitochondrial function and morphology. Mitochondrial function or the ability to generate energy through OXPHOS (oxidative phosphorylation) is vital for cell homeostasis and its dysfunction has been linked to the pathogenesis of nearly all chronic diseases (Pieczenik and Neustadt, 2007). Relevant to the studies presented in this manuscript, mitochondrial dysfunction is a key contributor to the perpetuation of metabolic inflammation caused by nutrient excess. This is accomplished, in part, through the inability to utilize excess fatty acids through inhibition of enzymatic function and the generation of excessive oxidative stress (Hotamisligil, 2006; Gregor and Hotamisligil, 2011; Satapati et al., 2015). Besides OXPHOS, mitochondrial dysfunction is also present in the examination of mitochondrial morphology, the balance of mitochondrial network formation, and fission and fusion.

Excessive mitochondrial fission is linked to mitochondrial dysfunction and reductions in OXPHOS (Wai and Langer, 2016) whereas inhibition of mitochondrial fission lessens pathological phenotypes in several experimental disease models such as; myocardial infarction (Disatnik et al., 2013), cardiac arrest (Sharp et al., 2015), acute cardiorenal syndrome (Sumida et al., 2015), glaucoma (Kim et al., 2015), neuronal ischemia (Grohm et al., 2012), traumatic brain injury (Fischer et al., 2016; Wu et al., 2016), anesthesia-induced neuronal injury (Xu et al., 2016), and multiple models of neurodegeneration (Reddy, 2014). These data demonstrate that restoring the balance between fission and fusion may be a viable therapeutic approach.

To this end, we employed both *in vitro* cell culture and *in vivo* animal experimental models to study the therapeutic impact of human-derived MSCs. Initially, skin fibroblasts from patients with mitochondrial disease were co-cultured with human-derived MSCs and their mitochondrial morphology examined as a surrogate of mitochondrial dysfunction (Giedt et al., 2016). Secondly, metabolic inflammation was induced in animals following a prolonged high-fat feeding regiment before being treated with a single dose of intravenously administered human-derived MSCs for 24 h. Previous work from our laboratory shows that mouse-derived MSCs can improve the host metabolic capacity in a model of diet induced metabolic inflammation (Nyamandi et al., 2013). Given these data, our next question was to specifically explore the impact of human-derived MSC administration on mitochondrial function and morphology in host tissues. Mitochondrial metabolism, microarray gene expression profiling and metabolomic profiling of host liver tissue were examined. The liver was chosen as our tissue of interest because of its inherent ability to attract stem cells caused by lipid accumulation, its high concentration of mitochondria, and the presentation of metabolic inflammation following prolonged high-fat feeding (Ren et al., 2012; Zhao et al., 2015). Our hypotheses were two-fold: (1) that MSC contact co-culture would alter the mitochondrial morphology of skin fibroblasts from mitochondrial disease patients, and (2) that human-derived MSC therapy would promote cell signaling within mouse liver tissue, culminating in improved mitochondrial function, altered gene expression profiles, and differences in host metabolites.

## Materials and methods

### Study approval

This study was carried out in accordance with the recommendations of the University of Calgary Conjoint Health Research Ethics Board's recommendations with written informed consent from all subjects. Each subject gave written informed consent in accordance with the Declaration of Helsinki and the study protocol was approved by the University of Calgary's Conjoint Health Research Ethics Board (REB13-0753). This study was carried out in accordance with the recommendations of the Canadian Council on Animal Care and the University of Calgary Animal Care and Use Committee. The protocol, under the ethics report (BI11R-29), was approved by the University of Calgary Animal Care and Use Committee.

### Skin fibroblast and MSC culture

Four patients (2M, 2F) aged 46–65 years of age were seen in the Metabolic Clinic at Alberta Children's Hospital (Calgary, AB) and investigated for mitochondrial disease (Table 1). Four controls (2M, 2F) aged 46–62 years of age were also selected from a bank of skin fibroblasts that had previously been investigated for and found not to have a diagnosis of an inborn error of metabolism or a mitochondrial disease through the Metabolic Clinic at Alberta Children's Hospital. Patient and control skin biopsy tissues were accessed from the cell bank at Alberta Children's Hospital before being passaged and expanded into T75 flasks as per the protocol established by the Biochemical Genetics Laboratory at Alberta Children's Hospital. Cellular media, comprised of minimum essential medium (MEM) with 2 mM glutamine (Life Technologies; Burlington, ON), 10% fetal bovine serum (FBS) (Life Technologies), 1 mM sodium pyruvate (Life Technologies), 20 mM uridine (Life Technologies), and 100 U/mL Penicillin-Streptomycin, was replaced after 3 successive days of cell incubation. Upon reaching confluence, skin fibroblasts were detached from their respective flasks using 3 mL 0.25% trypsin/1 mM EDTA (Life Technologies) and monitored under light microscopy to confirm successful separation. Detached skin fibroblasts were then prepared for either fixation and immunofluorescent staining (see section Immunofluorescent Staining) or re-plated for co-culture experimentation (see section Fluorescent Staining and Co-Culture). Con-currently, human bone marrow-derived MSCs were provided in kind from the University of Calgary Cellular Therapy Laboratory (Dr. Nicole Prokopishyn). MSCs were cultured using the same protocol as listed above and were grown to confluency before being prepared for co-culture experimentation. All experiments were completed using skin fibroblasts and MSCs from passages 4 to 6.

**Table 1:** Subject mtDNA mutations.

**ID**	**Age**	**mtDNA mutation**
C1	60–64	None
C2	60–64	None
C3	45–49	None
C4	50–54	None
P1	50–54	m.8753_16566
P2	60–64	ATPase_CytB
P3	45–49	m.9090_16070
P4	60–64	m.9928, ATPase6

*Patient (P) and control (C) diagnoses of pathogenic mtDNA mutations were confirmed by genetic sequencing. Each cohort was comprised of two males and two females. Data were provided by the Molecular Diagnostics Laboratory at the University of Alberta (Edmonton, AB—courtesy of Dr. Stacey Hume)*.

### Immunofluorescent staining

Detached skin fibroblasts were counted (MOXI Z Mini Automated Cell Counter; ORFLO Technologies, Ketchum, ID) and seeded on three autoclaved coverslips per subject at 5.0 × 10^4^ cells per slip using a 24 well-plate. Cells were incubated at 37°C overnight before being fixed with 4% paraformaldehyde in phosphate buffered saline (PBS) at 37°C for 15 min. Coverslips were quenched with NH_4_Cl for 15 min and washed with PBS to minimize autofluorescence. Cells were permeabilized with 0.5 mL 0.2% Triton X-100 (Sigma; Oakville, ON) in PBS for 15 min, washed with PBS, and then blocked with 10% FBS (Life Technologies) in PBS for 30 min. Cover slips were simultaneously incubated with primary antibodies for the outer mitochondrial membrane protein TOMM20 (FL-145, Santa Cruz; Dallas, TX) and DNA (CBL-186, EMD Millipore; Etobicoke, ON) diluted to 1:1,000 in 5% FBS, 95% PBS (Life Technologies) at 37°C for 1 h. Cells were washed 3 x with PBS and then incubated with secondary antibodies (1:5,000) at RT for 1 h. The following secondary antibodies conjugated to fluorescent dyes were used: Alexa Fluor 488 goat anti-mouse IgG (TOMM20, Molecular Probes, Eugene, OR) and Alexa Fluor 647 goat anti-rabbit IgG (DNA, Molecular Probes). Cells were mounted with DakoCytomation fluorescent mounting medium (Agilent Technologies; Santa Clara, CA) and stored at 4°C until microscopic examination.

### Fluorescent staining and co-culture

Detached skin fibroblasts were counted (MOXI Z Mini Automated Cell Counter) and seeded at 7.5 × 10^4^ cells on 35 mm dishes with embedded, uncoated glass coverslips (MatTek; Ashland, MA) and incubated overnight. Live cells were fluorescently labeled with MitoTracker Deep Red FM (Thermo Fisher Scientific; Waltham, MA) for mitochondria and Quant-iT PicoGreen dsDNA (Thermo Fisher Scientific) for nDNA and mtDNA as per the manufacturer's instructions. Separately, MSC's were cultured and fluorescently labeled with MitoTracker Red CMXRos (Thermo Fisher Scientific) for mitochondria per the manufacturer's instructions. Following 1 h of incubation, labeled MSCs were harvested and counted (MOXI Z Mini Automated Cell Counter) before being co-cultured with existing skin fibroblast cultures at 7.5 × 10^4^ cells per 35 mm dish. Co-cultures were imaged live after an 8-h co-culture period, with conditions replicated for each subject.

### Confocal microscopy and mitochondrial morphology

Mitochondrial morphology and mtDNA nucleoid structure were analyzed by the appropriate lasers using a confocal scanning microscope (Zeiss LSM 700, Carl Zeiss Microscopy; Jena, Germany). All images were collected using a 2,048 × 2,048-pixel configuration of single confocal planes. Each image was acquired and processed using the accompanying ZEN software, version 2.3 (Carl Zeiss Microscopy). Both fixed individual and live co-cultured skin fibroblast cells were manually classified into one of five mitochondrial morphology categories and one of two categories for mtDNA structure. Our classification system was developed to further describe the fission and fusion morphologies as previously described (Sabouny et al., 2017). At least 150 cells from each condition were counted from multiple independent experiments and scored by eye for morphology into the indicated classes. Morphology and mtDNA structure were evaluated by blinded investigators by comparing to a set of standard images (Figure S1).

### Animals and dietary interventions

Male C57BL/6 mice were housed in a humidity and temperature (21–22°C) controlled room with a 12 h light/dark Zeitgeber cycle and free access to both food and water. At 4 weeks of age, animals were placed on a high-fat diet (HF, 60% kcal fat; D12492, Research Diets; New Brunswick, NJ) for 20 weeks in order to elicit metabolic inflammation (Guo et al., 2009). Following dietary manipulation, animals were randomly divided into two groups: MSC therapy (HFM; *n* = 8) or saline control (HFS; *n* = 8).

### MSC therapy

Fluorescently labeled (GFP) human bone marrow-derived MSCs were purchased from the Center for the Preparation and Distribution of Adult Stem Cells (Institute for Regenerative Medicine, TX), an NIH/NCRR (P40 RR 17447-06) supported MSC distribution center. Delivered MSCs have been shown to successfully differentiate into adipocytes, chondrocytes, and osteocytes as well as possess the following hematopoietic markers: CD34^−^, CD36^−^, CD117^−^, CD45^−^, CD29^+^, CD44^+^, CD49c^+^, CD49f^+^, CD59^+^, CD90^+^, CD105^+^, and CD166^+^ (Oh et al., 2012). Cells were passaged and expanded per the same protocol as described previously. Upon reaching confluence, MSCs were detached from their respective flasks using 3 mL 0.25% trypsin/1 mM EDTA (Life Technologies) and monitored under light microscopy to establish successful separation. Detached MSC were then purified through three stages of centrifugation and counted using a hemocytometer before being suspended in PBS at a density of 7.5 × 10^5^ cells per 0.2 ml. Following dietary intervention, animals were injected with equal volumes (0.2 mL) of 7.5 × 10^5^ MSCs or PBS via the tail vein. All experiments were completed using MSCs from a single donor and passages 4–6. Twenty-four hours post-injection the animals were weighed and then sacrificed via cervical dislocation. Tissues (liver and whole blood) were removed and either kept fresh or flash frozen and stored at −80°C for future testing.

### Sequence-specific qualitative PCR

Total genomic DNA was extracted from 25 mg of frozen liver tissue using the QIAamp DNA Mini Kit (Qiagen; Germantown, MD). DNA concentrations were measured using a NanoDrop-1,000 (Thermo Fisher Scientific). Distinct human and mouse-specific primer pairs were used to distinguish effective administration of MSCs, as documented previously (Alcoser et al., 2011), with primers synthesized by the University of Calgary Core DNA Services (Table S1). A positive human control was obtained from a cultured fibroblast cell line. PCR was performed using undiluted genomic DNA (150–200 ng) on a Mastercycler ep Gradient S (Eppendorf; Hamburg, Germany). PCR conditions were as follows: 95°C−5 min, 30 cycles of (94°C−45 s, 60°C−30 s, 72°C−90 s), 72°C−10 min. Amplified PCR products were run undiluted on a 2% agarose gel plus ethidium bromide (0.5 μg/ml).

### Mitochondrial respirometry

Fresh liver tissue from the upper left lobe was rinsed and homogenized in liver mitochondria isolation buffer (250 mM sucrose, 2 mM KH_2_PO_4_, 1 mM EGTA, 20 mM Tris-HCl, pH 7.4 at 4°C) (Sigma) before undergoing differential centrifugation, according to previous procedures with slight variations (Frezza et al., 2007). Mitochondrial protein quantification was performed with bovine serum albumin as the standard using the Bradford method (Bio-Rad). High-resolution respirometry measurements were then performed on isolated mitochondria samples using the Oroboros Oxygraph-2k (Oroboros Instruments; Innsbruck, Austria) at 37°C in MiR05 (0.5 mM EGTA, 3 mM MgCl_2_centre.6H_2_O, 20 mM taurine, 10 mM KH_2_PO_4_, pH 7.1) (Sigma). Each sample was run in duplicates to ensure repeatability of results. Substrate additions were as follows (final concentration): 10 mM glutamate plus 0.5 mM malate, 2 mM ADP, and 2 μg/ml oligomycin (Sigma). A final addition of 10 mM cytochrome c was added to establish the intactness of the mitochondrial outer membrane (Figure S2).

### Mitochondrial enzyme activities

Citrate synthase activity, a mitochondrial enzyme and marker of mitochondrial content, was measured from liver homogenates using a spectrophotometric method (Tweedie et al., 2011). Total liver superoxide dismutase (SOD) activity was assessed as previously described, with slight variations (Gianni et al., 2004). Liver homogenate protein quantification was performed with bovine serum albumin as the standard using the Bradford method (Bio-Rad; Des Plaines, IL). Procedures were performed on a DU 800 Spectrophotometer (Beckman Coulter; Mississauga, ON) with data normalized to mg protein.

### Liver mitochondrial ROS production

Fresh liver tissue from the upper right lobe was rinsed, homogenized and centrifuged in order to isolate hepatic mitochondria (Frezza et al., 2007). Mitochondrial protein quantification was performed with bovine serum albumin as the standard using the Bradford method (Bio-Rad). Liver mitochondrial reactive oxygen species (ROS) production was measured using fluorometry (Hitachi F-2700 Spectrophotometer, Hitachi America; Elk Grove Village, IL) according to previous research (Nyamandi et al., 2013).

### Microarray gene expression profiling

Expression profiling was performed as described previously (Hittel et al., 2003), using 5 μg of total RNA extracted from frozen liver tissue from animals in both groups (*n* = 7 per group due to damage of one sample per group in transit) (Hittel et al., 2010). Biotinylated cRNA was synthesized from cDNA and fragmentation as detailed in the manufacturer's protocol for GeneChip Mouse Gene 1.0 ST microarrays (Affymetrix; Santa Clara, CA). Stringent quality control methods were employed (Hittel et al., 2005). Each array fulfilled the following quality control measures: average cRNA fold changes of 15.6, average scaling factor of 3.9 to a target intensity of 500, average “present” calls 43%, showed >80% present calls, consistent values across samples, average 5′/3′ ratios of housekeeping genes, and internal probe set controls was 0.81 (Hubal et al., 2011). All microarray data are to be published and freely accessible at NCBI-GEO (GSE121879).

### RNA extraction and qRT-PCR

Total RNA was extracted from 25 mg of frozen liver tissue using the PureLink RNA Mini Kit (Life Technologies) and was measured using a NanoDrop-1000 (Thermo Fisher Scientific). Reverse transcription was performed with 1 μg of RNA using the iScript cDNA Synthesis Kit (Bio-Rad). cDNA products were quantified using a NanoDrop-1000 (Thermo Fisher Scientific). qRT-PCR was performed in 20 μl reaction volumes using 50 ng of cDNA. Representative genes, 3 upregulated and 3 downregulated, were randomly selected for measurement using qRT-PCR to verify DNA microarray results. qRT-PCR primers were synthesized by the University of Calgary Core DNA Services (Table 1). The qRT-PCR conditions were as follows: 95°C−2 min, 40 cycles of (95°C−30 s, 60°C−30 s, 72°C−30 s), 72°C−2 min. Samples were run in triplicate on the same reaction plate using the CFX96 Real-Time PCR Detection System (Bio-Rad) with β-actin as a loading control. Data analysis was performed in accordance with previously published work using the 2^−ΔΔ*Ct*^ method (Lee et al., 2012).

### Metabolomics sample preparation

Liver samples were prepared according to previously published protocols (Wu et al., 2008). Briefly, frozen samples of roughly 100 mg were homogenized in a Fastprep-24 homogenizer (MP Biomedicals; Santa Ana, CA) using 1.4 mm ceramic beads (“Lysing Matrix D,” MP Biomedicals). Before homogenization, 400 μL cold methanol and 85 μL water were added. After homogenization, 400 μL chloroform and 200 μL water were added and the samples kept on ice for 10 min. Samples were centrifuged for 5 min at 2,000 × g at 4°. The aqueous and the lipophilic layer were collected separately and evaporated in a Vacufuge Concentrator 5301 (Eppendorf). Evaporated aqueous extracts were dissolved in 400 μL water and mixed with 200 μL buffer containing TSP and 50 μL deuterium oxide. Evaporated lipophilic extracts were dissolved in deuterated chloroform containing octamethylcyclotetrasiloxane (OMS) as internal standard (Thomas et al., 2012).

### Metabolomics NMR measurements

1D ^1^H NOESY spectra were measured at 298 K on an Avance II 600 MHz NMR spectrometer equipped with a triple-resonance probe, z-gradients, and an automatic sample changer (Bruker BioSpin; Milton, ON). Spectra of aqueous liver extracts were collected using water presaturation and spoil gradients for water suppression. Acquisition and processing parameters were chosen as published before (Gronwald et al., 2008).

### Metabolomics data preparation

NMR spectra of aqueous extracts were split into equally sized bins of 0.01 ppm width in the range of 0.5–9.5 ppm using AMIX version 3.9.15 (Bruker BioSpin). Regions containing water and methanol were excluded from binning. Bin intensities were scaled to the TSP signal and then corrected for sample weight. Bins with a mean intensity of <3.5 times the noise level were excluded from further analysis. Lipid signals were integrated from the lipophilic liver extracts relative to the OMS peak and then corrected for sample weight (Klein et al., 2010a). Signals corresponding to saturated, unsaturated, and polyunsaturated fatty acid bonds, as well as total cholesterol were integrated for further analysis. Total lipids, Saturation Index (SI), Unsaturation Index (UI), Polyunsaturation Index (PUI), and Polyunsaturated Fatty Acids/Monounsaturated Fatty Acids (PUFA/MUFA) were calculated as described before (Klein et al., 2010b).

### Statistical analyses

Statistical analysis for mitochondrial morphology, animal characteristics, respirometry, mitochondrial enzyme activity, and ROS production was performed using GraphPad Prism for Windows, Version 7.01 (GraphPad Software Inc.; La Jolla, CA). Differences between treatment groups were determined by Student's unpaired, two-tailed *t*-tests where *p* < 0.05 was significant. Data are expressed as mean ± SEM. For microarray data, expression values for probe sets were generated from the probe logarithmic intensity error (PLIER) algorithm in Expression Console (Affymetrix) and imported directly into the Partek Genomics Suite, version 6.2 (Partek; St. Louis, MO) for statistical processing. Microarray metadata complied with MIAME standards, and all samples will be submitted to the NCBI GEO database for public access. All outputs were filtered with a *p* < 0.01 cut-off. Following these analyses, a Bonferroni multiple testing correction was applied to reduce the false positive rate. Apart from data sets uploaded into pathway generation software, a 1.2-fold change filter was also applied. Lists of probe sets passing the *p*-value filter for differential expression between genotype groups were imported into Ingenuity Pathway Analysis software, version 7.5 (Ingenuity Systems; Redwood City, CA), for network and pathway generation. In order to examine common pathways, the Database for Annotation, Visualization and Integrated Discovery (DAVID) was employed (Huang et al., 2009a,b). All statistical analyses of metabolite data were performed in *R* version 3.2.3. Metabolic differences were assessed using Mann-Whitney U-tests including False Discovery Rate (FDR) controlling. Significance was assumed for FDR values below 20%.

## Results

### Co-culture of patient skin fibroblasts with MSC rescues aberrant mitochondrial morphology

**Figure 1:**
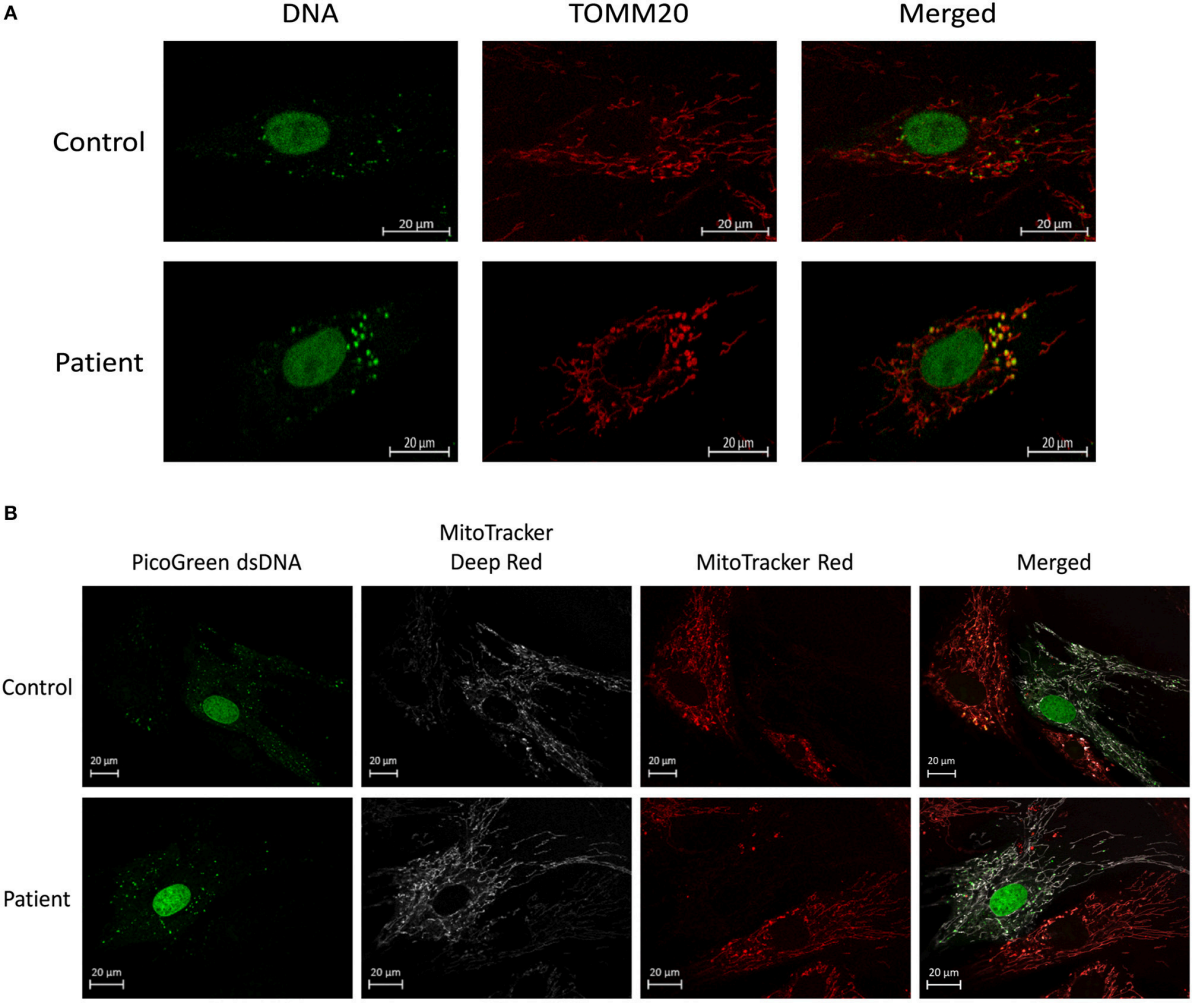
Mitochondrial morphology and mtDNA of fixed fibroblasts and live fibroblasts co-cultured with MSCs. **(A)** Representative images demonstrating assessment of mitochondrial morphology and mtDNA of fixed fibroblasts from controls and patients with clinically diagnosed mitochondrial disease. Images were collected using immunofluorescence against TOMM20 (mitochondria) and DNA (DNA). **(B)** Representative images demonstrating assessment of mitochondrial morphology and mtDNA of live fibroblasts (from controls and patients with a clinically diagnosed mitochondrial disease) co-cultured with MSCs. Images were collected following fluorescent labeling of fibroblasts with MitoTracker Deep Red (mitochondria) and PicoGreen (DNA). MSCs were separately fluorescently labeled with MitoTracker Red (mitochondria) prior to co-culture. Images from both panels were collected using confocal microscopy.

**Figure 2:**
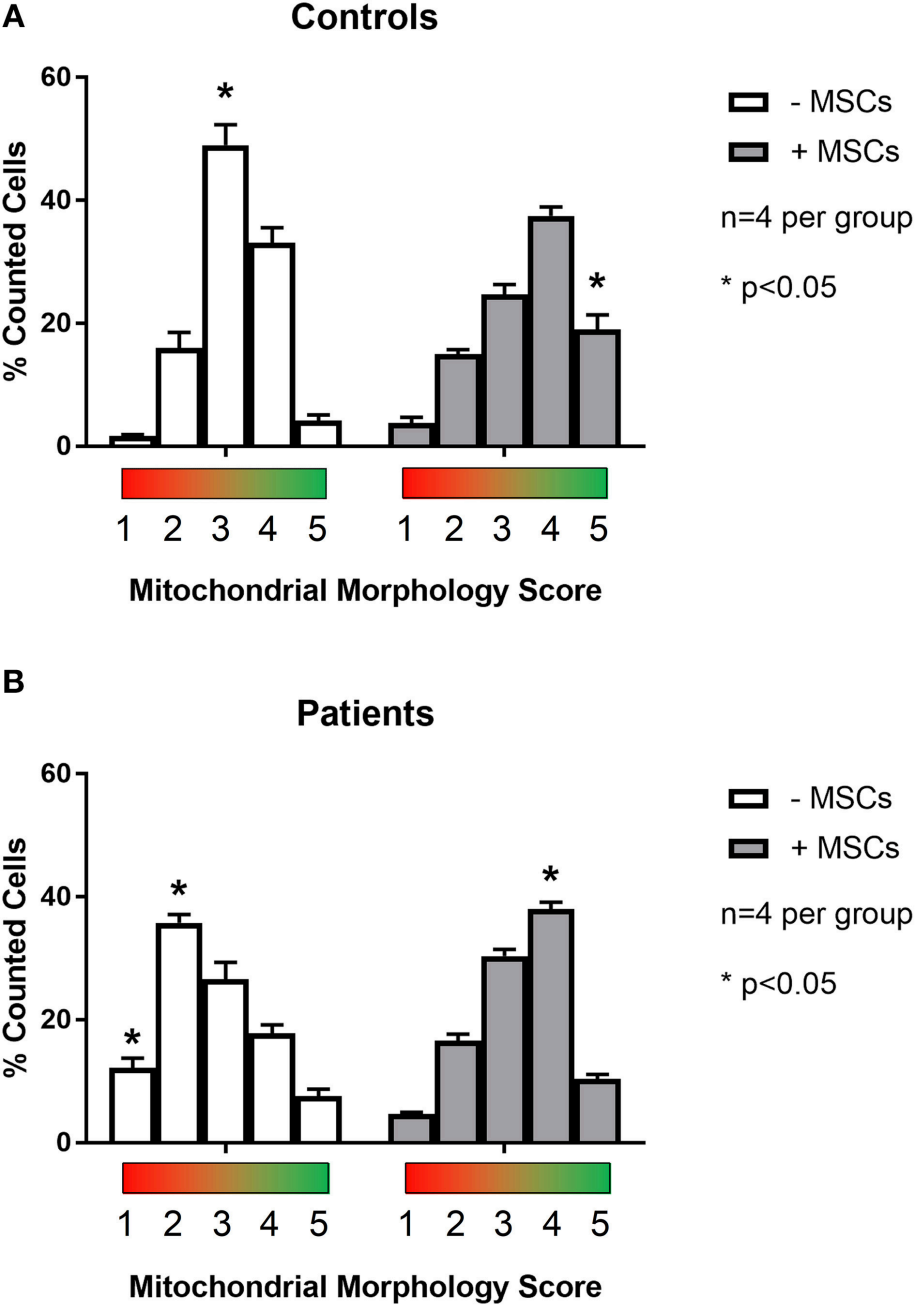
Quantification of mitochondrial morphology scores between patient and control cells. Skin fibroblasts from healthy controls **(A)** and patients with a clinically diagnosed mitochondrial disease **(B)** were manually classified into one of five mitochondrial morphology categories. Category 1 (red) corresponds to a fully fragmented morphology and category 5 (green) corresponds to a fusion morphology. Results from baseline were compared following contact co-culture with mesenchymal stem cells (MSCs). A minimum of 150 cells were quantified per metric and per condition from 2 to 3 independent experiments. All data are mean ± SEM with *n* = 4 for each group. ^*^Denotes statistical significance at *p* < 0.05.

**Figure 3:**
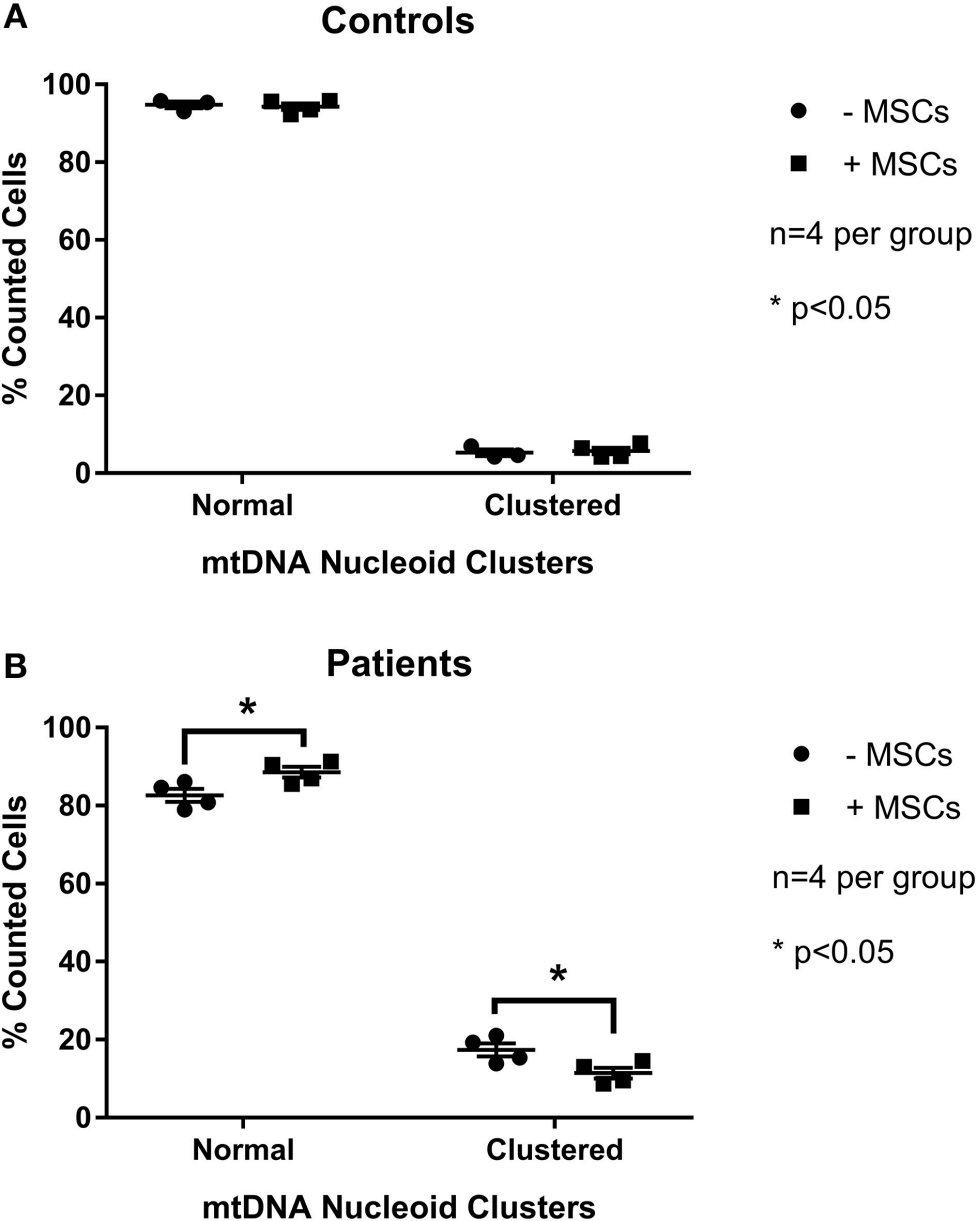
Quantification of mitochondrial mtDNA nucleoid clusters between patient and control cells. Skin fibroblasts from healthy controls **(A)** and patients with a clinically diagnosed mitochondrial disease **(B)** were manually classified into one of twocategories for mtDNA structure. Results from baseline were compared following contact co-culture with mesenchymal stem cells (MSCs). A minimum of 150 cells were quantified per metric and per condition from 2 to 3 independent experiments. All data are mean ± SEM with *n* = 4 for each group. ^*^Denotes statistical significance at *p* < 0.05.

To examine the effect of MSCs using an *in vitro* model of mitochondrial dysfunction (fission morphology), skin fibroblasts from patients with long-standing, well-characterized mitochondrial disease, and matched healthy controls underwent contact co-culture with MSCs. As opposed to traditional measures of mitochondrial function (i.e., OXPHOS), mitochondrial morphology was studied since each cell population could be separately identified following staining and visualization in real-time. Mitochondrial morphology was classified into one of five categories ranging from fragmented to hyper-fused and mitochondrial DNA (mtDNA) structure into normal or clustered categories (Figure S1). The mitochondrial morphology of patient cells was significantly more fragmented than healthy controls, demonstrating a fission morphology (Figures 1, 2; *p* < 0.05). Fission morphology has been well-documented in models of mitochondrial disease and is hypothesized to influence human disease pathologies through mediation of: low ATP production, low-grade inflammation, and mitochondrial network fragmentation (Archer, 2013). It is also consistent with previous cell culture knockdown models where excessive fission induces severe cellular defects and decreased ATP levels (Jheng et al., 2012). When comparing mtDNA structure there was a higher prevalence of mtDNA clusters in patient cells (Figure 3; *p* < 0.05). The aggregation of mtDNA into nucleoid clusters has been studied as a physiologic response to mtDNA stressors (Alán et al., 2016). Following MSC contact co-culture there was a rescue of the initial fission morphology, shifting patient mitochondria to an elongated fusion pattern (Figure 2; *p* < 0.05). Furthermore, there was a 34% decrease in the percent of patient cells with clustered mtDNA nucleoids following MSC co-culture (Figure 3; *p* < 0.05). Live cell imaging was also performed to capture transfer of labeled mitochondrial components from MSCs to fibroblasts. As identified in previously published work, bi-directional transfer of labeled mitochondrial components was identified (Shi et al., 2010; Bernardo and Fibbe, 2012; Islam et al., 2012) (Video S1). Together, these results indicate MSCs induce morphologic shifts in a cell culture model of mitochondrial dysfunction, and that this may be a result of bi-directional mitochondrial transfer.

### Mitochondrial function is improved following MSC therapy

In the next set of experiments, we sought to examine mitochondrial function with MSC administration in a model of impaired mitochondrial respiration, as opposed to overt mitochondrial disease resulting from genetic mutation. To this end, we chose a sub-pathological state of mitochondrial dysfunction by exposing animals to prolonged high-fat feeding (20 week), a well-characterized model known to perturb mitochondrial function (Anderson et al., 2009; Nyamandi et al., 2013). MSC were intravenously administered via the tail vein for a 24 h period prior to sacrifice.

**Figure 4:**
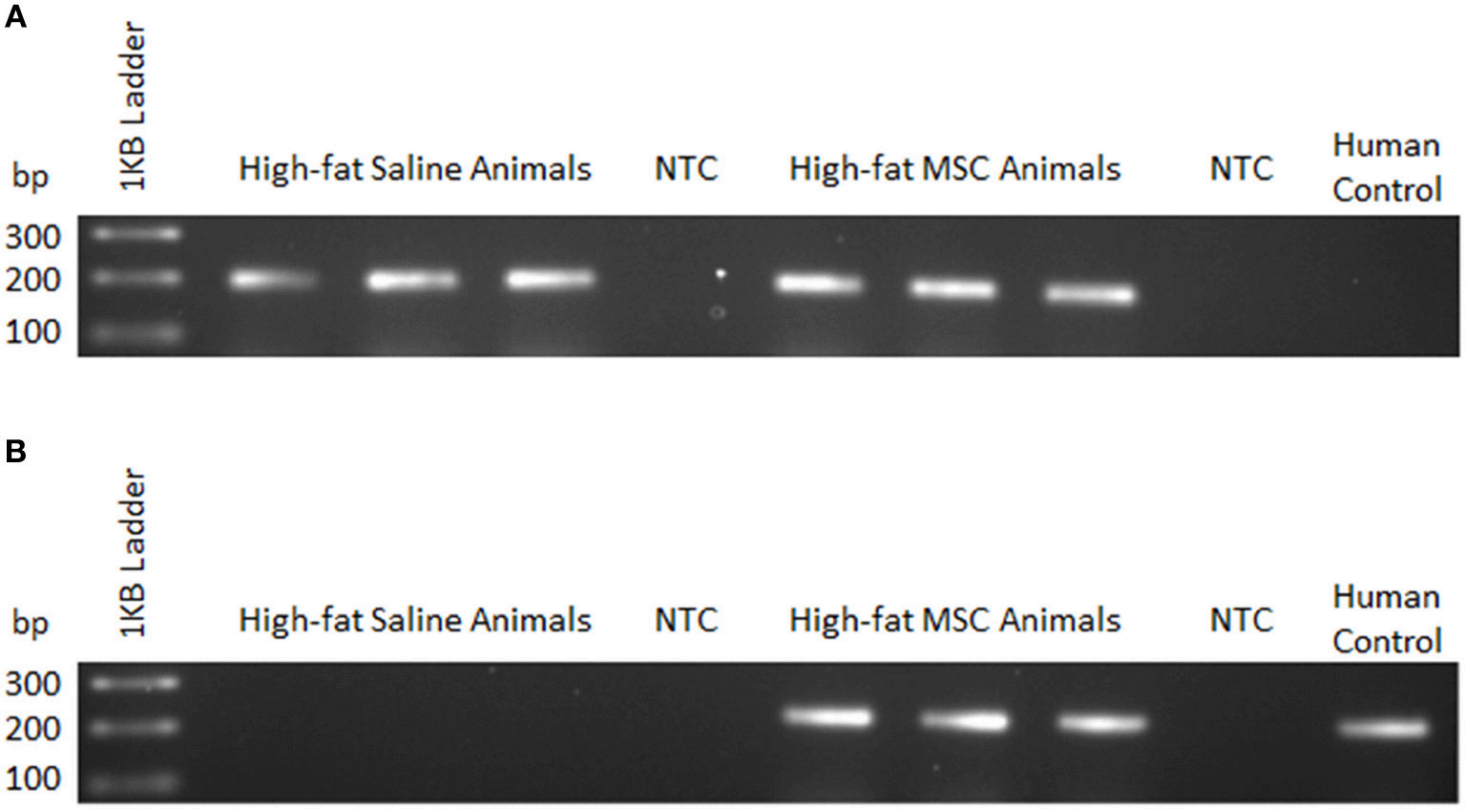
MSC detection using sequence-specific qualitative PCR. Liver tissue homogenates were used to detect mouse and human genomic DNA 24 h following control (saline) or MSC therapy into C57BL/6 mice. **(A)** Mouse—forward and common reverse PCR primers for prostaglandin E receptor 2 (PTGER2) were amplified to detect the presence of mouse-specific genetic material. **(B)** Human—forward and common reverse PCR primers for a non-homologous region of PTGER2 were amplified to detect the presence of human-specific genetic material. Human control samples were isolated from a cultured human cell line. NTC, no template control.

To confirm MSC presence following transplantation, qualitative PCR was used to detect sequence-specific genomic DNA in the liver. This method was employed due to complications in distinguishing fluorescently labeled MSCs from autofluorescence of liver tissue during confocal imaging following quenching of tissue. Appropriately, PCR primers specific to the mouse sequence of prostaglandin E receptor 2 (PTGER2) confirmed the presence of genomic mouse DNA in both high-fat saline treated (HFS) and high-fat MSC treated (HFM) groups (Figure 4A). Human specific PCR primers for a non-homologous region of the PTGER2 gene (Alcoser et al., 2011) were only successful in amplifying genetic material from HFM samples, providing evidence that MSC were only present within the liver tissue of MSC treated animals (Figure 4B).

To examine mitochondrial function with MSC treatment, specific mitochondrial complex activity was assessed by high-resolution respirometry. Evaluation of respirometry data, for both basal oxygen consumption supported by glutamate and malate, oligomycin mediated proton leak (a state 4 condition) and the respiratory control ratio (RCR; State 3/State 4), a measure of mitochondrial oxidative coupling efficiency, was similar between groups (Table 2; *p* < 0.05). However, maximal complex I oxygen consumption as assessed by the addition of ADP (a state 3 condition), was higher in HFM animals by 46% (22.4 ± 1.0 vs. 32.8 ± 3.8, *p* < 0.05). Next, we sought to explain these alterations in oxidative metabolism by measuring citrate synthase enzyme activity, a marker of mitochondrial abundance. High fat fed animals treated with MSCs demonstrated a 1.5-fold increase in citrate synthase enzyme activity compared to controls, indicating a mechanism responsible for the oxidative changes (15.5 ± 1.0 vs. 23.7 ± 1.1, μm/min/mg protein liver *p* < 0.05). Taken together, these findings indicate that MSC administration increases both maximal oxidative capacity and mitochondrial abundance.

**Table 2:** Mitochondrial respirometry performed on mitochondria isolated from liver tissue homogenates.

	**High-fat**	
	**Saline**	**MSC**
Glutamate + Malate	2.4 ± 0.1	3.0 ± 0.5
ADP	22.4 ± 1.0	32.8 ± 3.8^*^
Oligomycin	2.6 ± 0.1	3.4 ± 0.5
RCR	9.4 ± 0.6	10.0 ± 1.0
Citrate synthase activity	15.5 ± 1.0	23.7 ± 1.1^*^

Basal oxygen consumption supported by glutamate and malate through complex I (Glutamate + Malate), maximal complex I oxygen consumption (ADP), oligomycin mediated proton leak (Oligomycin), and respiratory control ratio (RCR), a measure of mitochondrial oxidative coupling efficiency. Respirometric data was normalized to mg of mitochondrial protein. Data are mean ± SEM with n = 8 for both groups.

* *Denotes statistical significance at p < 0.05*.

### Enhanced ROS production at complex III with MSC administration

**Figure 5:**
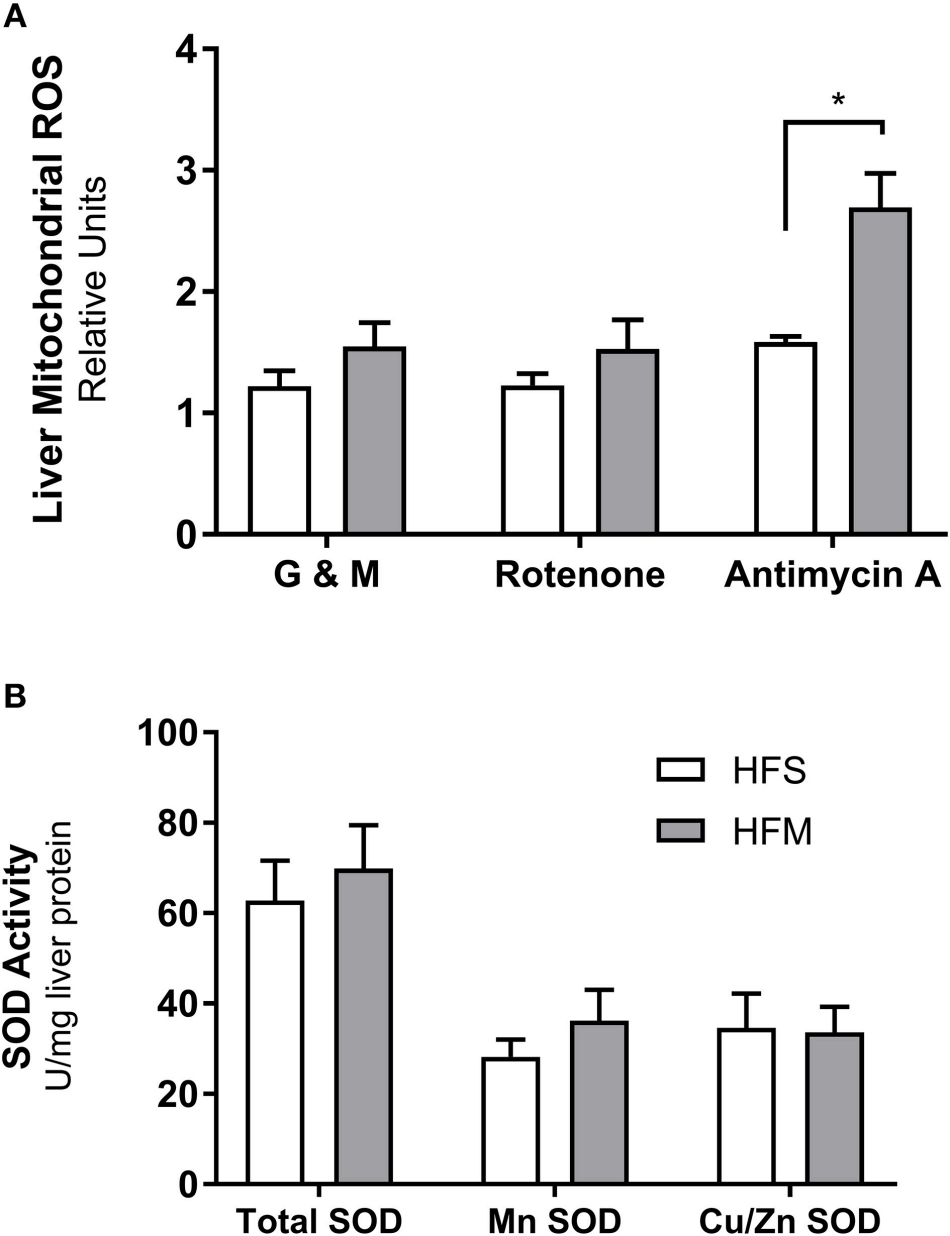
Detection of reactive oxygen species production and superoxide dismutase enzyme activity from liver homogenates. Following control (saline) or MSC therapy, liver homogenates were used to quantify the generation of reactive oxygen species (ROS) and the free radical scavenging enzyme superoxide dismutase (SOD). **(A)** Relative rates of H_2_O_2_ production as a function of ROS generation. Mitochondria isolated from liver tissue were stimulated in the presence of ADP under a variety of conditions including: glutamate and malate as substrates (G & M; complex I), rotenone as an inhibitor (complex I), and antimycin as an inhibitor (complex III). **(B)** SOD enzyme activity measured from liver homogenates. SOD activity is stratified into manganese (Mn) or zinc and copper (Zn and Cu) fractions, with total representing both portions combined. SOD data are normalized to mg of liver protein and ROS data are normalized to mg of mitochondrial protein. All data are mean ± SEM with *n* = 8 for both groups. ^*^Denotes statistical significance at *p* < 0.05.

As a metabolic by-product to mitochondrial metabolism, ROS have been implicated as both a molecule involved in healthy cell signaling, and as a damaging source of oxygen free radicals responsible for oxidative stress and mitochondrial dysfunction (Zorov et al., 2014). To assess the changes associated with mitochondrial abundance and oxidative capacity, we examined levels of oxidative stress by measuring H_2_O_2_ generation from liver mitochondrial isolates (Figure 5A). Neither complex I mediated respiration (a state 3 condition) in the presence of ADP or rotenone inhibition caused altered H_2_O_2_ production between groups. However, adding complex III inhibitor and mitochondrial membrane depolarization agent antimycin A caused increased ROS production in HFM animals (*p* < 0.05) indicating that MSC treatment elevated mitochondrial membrane potential (Rego et al., 2001). To determine if MSC therapy simply increased the capacity of the liver to sequester excess superoxide anion production, we also studied SOD enzyme activity (Figure 5B). No differences were noted between total, cytosolic (Cu/Zn), or mitochondrial (Mn) SOD enzyme activity. The evidence of heightened ROS signaling and improved mitochondrial function suggests that healthy cell signaling regulation may be restored by MSC exposure.

### Widespread hepatic gene shifting following MSC therapy

To examine the effect of MSC therapy on global gene expression and cell signaling pathways, microarray gene expression analysis was performed on liver tissues. Results identified 226 genes with significant differential expression between groups (*p* < 0.01). Verification of microarray results was performed on selected genes using qRT-PCR from both upregulated and downregulated categories (Table 3). The direction and magnitude of fold change was consistent between both microarray and qRT-PCR results.

**Table 3:** Verification of microarray using qRT-PCR on genes randomly selected from both upregulated/downregulated categories.

**Systematic name**	**Gene symbol**	**Gene name**	**Fold change**	
			**Microarray**	**qRT-PCR**
**UPREGULATED**				
NM_009117	SAA1	Serum amyloid A 1	6.549
NM_008491	LCN2	Lipocalin 2	3.333	4.154
NM_008768	ORM1	Orosomucoid 1	3.071
NM_009690	CD5l	CD5 antigen-like	1.747
NM_010130	ADGRE1	Adhesion G protein-coupled receptor E1	1.624
NM_010745	LY86	Lymphocyte antigen 86	1.621
NM_011315	SAA3	Serum amyloid A 3	1.615	1.567
NM_008509	LPL	Lipoprotein lipase	1.582	1.989
NM_008533	CD180	CD180 antigen	1.576
**DOWNREGULATED**				
NM_206537	CYP2C54	Cytochrome P450, family 2, subfamily c, polypeptide 54	2.739
NM_010011	CYP4A10	Cytochrome P450, family 4, subfamily a, polypeptide 10	2.259
NM_007822	CYP4A14	Cytochrome P450, family 4, subfamily a, polypeptide 14	2.044
NM_009993	CYP1A2	Cytochrome P450, family 1, subfamily a, polypeptide 2	1.713
NM_010004	CYP2C40	Cytochrome P450, family 2, subfamily c, polypeptide 40	1.663
NM_012006	ACOT1	Acyl-CoA thioesterase 1	1.567	2.132
NM_026159	RETSAT	Retinol saturase (all trans retinol 13,14 reductase)	1.395	1.116
NM_011169	PRLR	Prolactin receptor	1.362	1.919
NM_019975	HACl1	2-Hydroxyacyl-CoA lyase 1	1.347

*All qRT-PCR data are presented as mean fold change with β-actin as a loading control. The corresponding fold change from microarray experiments is shown for reference. All data represent HFM vs. HFS (n = 7 for both groups)*.

**Figure 6:**
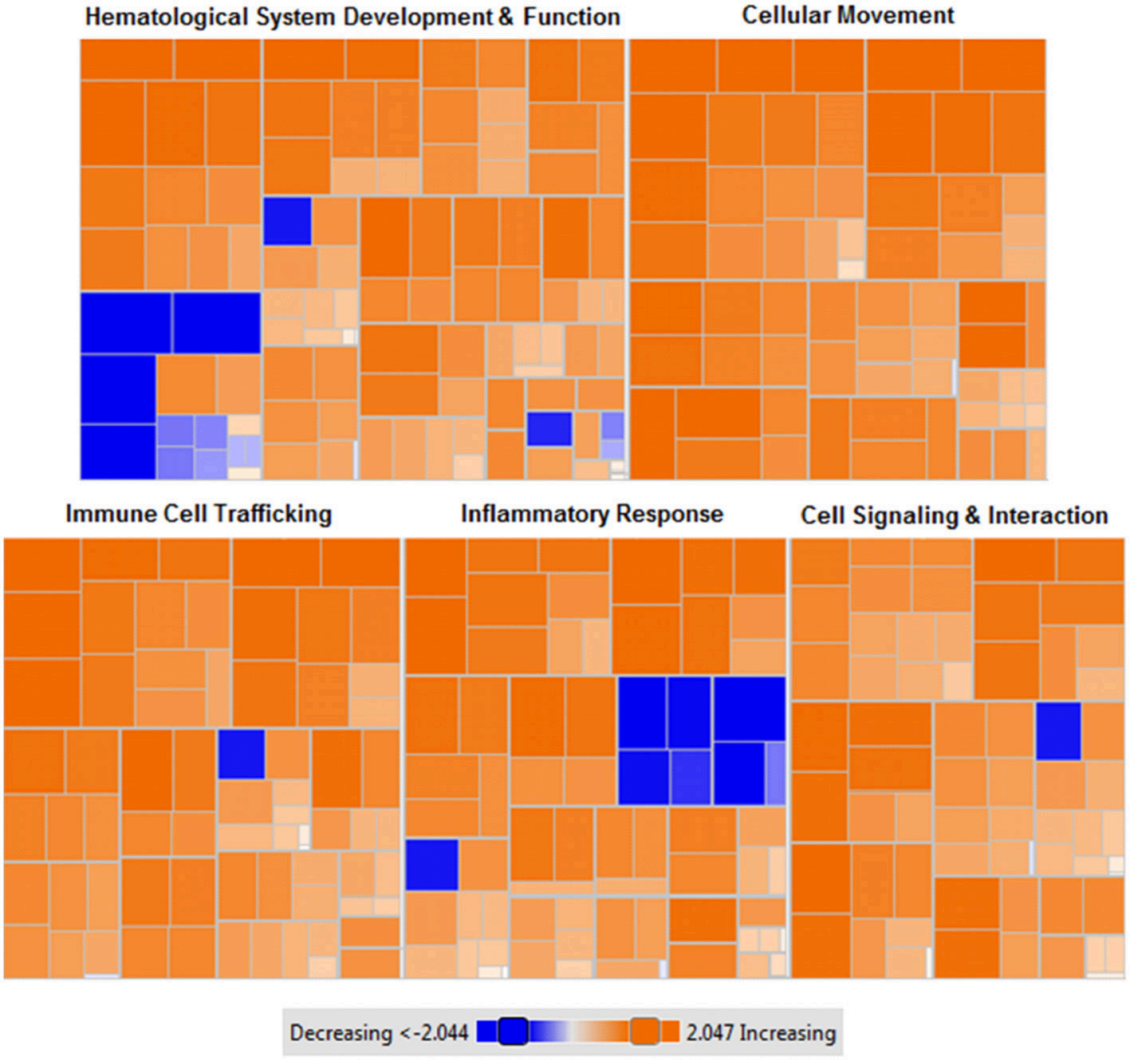
Heat map of functional categories of closely related genes with differential gene expression following MSC administration. Enriched gene ontology terms of MSC treated liver tissues compared to control (saline) liver tissues using z-scores computed from microarray gene expression profiling. Orange: upregulated gene expression (z-score >0), colorless: equal gene expression (z-score = 0), blue: downregulated gene expression (z-score <0). Data were generated from microarray gene expression data with each square corresponding to a single functional category. *n* = 7 tissues per condition. *p* < 0.001 for each (MSC treated vs. control).

Networks of closely related genes were also analyzed for differences following MSC therapy. These were then classified using common variations among genes utilizing functional categories derived from enriched gene ontology terms. Each of the five most variable categories contained a collective group of genes that were significantly upregulated in HFM liver samples (Figure 6; *p* < 0.001 for each category). These functional categories were further stratified as to describe their physiological function (Table 4). Cell-to-cell signaling and interaction was one gene network significantly elevated following MSC therapy (*p* < 0.05). Coinciding with our ROS data, these results show that cell signaling may be reinvigorated with MSC treatment through elevated ROS production.

**Table 4:** Functional categories of genes with differential expression in liver tissue following MSC therapy.

	**Function**	**Count**	***p*-value**	**Gene List**
A	Activation	30	3.29E^−9^-2.08E^−4^	ANXA2, AXL, BLK, C1QA, C4A/C4B, CD180, CD5L, CD68, CD72, COCO1C, CTSS, ELMO1, ENTPD1, ESM1, FCER1G, FCGR2A, FYB, GNAI2, HCK, HLA-A, HMOX1, IFNL3, ITGA4, ITGB2, LAPTM5, LCP2, LCN2, LCP2, LPL, LRP1, MARCO, MSN, MYO9B, PCSK9, PILRA, PLD4, PPP1R9B, RAC2, RARRES2, SAA1, SAA3, SIRPA, SOAT1, SOD3, TAP1, TYROBP, UCP2, UNC93B1, VCAM1, ZEB2
	Adhesion	20	1.84E^−8^-1.86E^−3^
	Binding	12	5.08E^−7^-1.53E^−3^
	Immune response	24	3.13E^−13^-1.08E^−3^
	Phagocytosis	18	5.22E^−9^-1.09E^−3^
	Recruitment	17	1.55E^−6^-1.42E^−3^
	Response	18	6.89E^−11^-3.67E^−4^
B	Cell movement	60	7.80E^−10^-1.04E^−3^	ALK, ANXA2, ARHGDIB, ARHGEF4, ARPC1B, AXL, C2, C4A/C4B, CD5L, CORO1C, CTSS, CYP1A2, CYP26C1, EAR2, EFEMP1, ELF4, ELMO1, ENTPD1, FABP4, FCER1G, FCGR2A, FYB, G6PD, GNAI2, GRB7, HCK, HLA-A, HMOX1, HP, IFNL3, ITGA4, ITGB2, LCN2, LCP2, LGMN, LRP1, MARCO, MIR-218, MSN, MYO9A, MYO9B, PLA2G7, PLXNA2, PPP1R9B, PRLR, RAC2, RARRES2, SAA1, SAA3, SDC3, SIRPA, SNAI2, SOD3, ST3GAL3, TGFBI, TXNDC2, TYROBP, UCP2, VCAM1, ZEB2
	Cellular infiltration	20	7.83E^−7^-1.54E^−3^
	Chemotaxis	23	2.38E^−7^-2.00E^−3^
	Homing	25	4.10E^−8^-9.31E^−4^
	Migration	55	1.74E^−9^-3.11E^−3^
	Recruitment	17	1.55E^−6^-3.29E^−3^
	Transmigration	8	4.05E^−5^-3.78E^−4^
C	Accumulation	10	3.91E^−4^-9.85E^−4^	AMBP, APOBEC1, ARHGDIB, AXL, BIRC3, BLK, C1QA, C4A/C4B, CD180, CD5L, CD72, CTSS, CYP4A11, ELF4, ELMO1, FCER1G, FCGR2A, FYB, GNAI2, GSX1, HCK, HLA-A, HMOX1, HPX, IFNL3, IRF2, ITGB2, LCN2, LCP2, LGMN, MYO9B, NLRC5, PILRA, RAC2, SAA1, SIRPA, SNAI2, SOAT1, TAP1, TGFBI, TYROBP, VCAM1
	Activation	30	3.29E^−9^-6.38E^−4^
	Adhesion	17	7.06E^−7^-1.31E^−3^
	Binding	11	2.93E^−6^-1.53E^−3^
	Cell movement	33	7.80E^−10^-8.45E^−5^
	Migration	15	3.24E^−6^-2.06E^−3^
	Quantity	42	7.70E^−13^-1.41E^−3^
D	Accumulation	10	2.29E^−5^-9.85E^−4^	ANXA2, C2, C4A/C4B, CD5L, CTSS, CYP1A2, EAR2, ELF4, ELMO1, FABP4, FCER1G, FCGR2A, FYB, GNAI2, HCK, HLA-A, HMOX1, HP, IFNL3, ITGA4, ITGB2, LCN2, LCP2, LRP1, MARCO, MYO9B, PLA2G7, RAC2, RARRES2, SAA1, SIRPA, SOD3, TYROBP, UCP2, VCAM1
	Activation	27	3.98E^−8^-6.38E^−4^
	Adhesion	17	7.06E^−7^-1.31E^−3^
	Cell movement	33	7.80E^−10^-8.45E^−5^
	Homing	19	7.21E^−8^-9.31E^−4^
	Migration	35	1.74E^−9^-2.06E^−3^
E	Accumulation	10	2.29E^−5^-9.85E^−4^	ADGRE1, AMBP, ARHGDIB, AXL, BIRC3, C1QA, C4A/C4B, CD180, CD5L, CD68, CD72, CTSS, ENTPD1, FABP4, FCER1G, FCGR2A, FRMD4B, GNAI2, GRB7, HCK, HLA-A, HMOX1, HP, HPGDS, HPX, IFNL3, IRF2, ITGA4, ITGB2, LCN2, LCP2, LGMN, LPL, MSN, PON1, SOAT1, ST3GAL3, TYROBP, UNC93B1, VCAM1
	Activation	27	3.98E^−8^-6.38E^−4^
	Immune response	31	3.13E^−13^-1.08E^−3^
	Inflammation	38	1.13E^−7^-2.89E^−4^
	Phagocytosis	20	8.19E^−10^-1.53E^−3^

*(A) Cell-to-cell signaling and interaction. (B) Cellular movement. (C) Hematological system development and function. (D) Immune cell trafficking. (E) Inflammatory response. Each category is stratified into collections of genes based on their proposed physiological function. Data were generated from gene microarray data and represent HFM vs. HFS (n = 7 for both groups)*.

To identify common pathways impacted by MSC therapy, data were also analyzed using the Kyoto Encyclopedia of Genes and Genomes Pathway Database (Kanehisa et al., 2016). Using this tool, functional annotation was performed to identify primary up and down activated pathways. Analysis revealed actin cytoskeleton regulation as the primary pathway activated by MSC administration (*p* < 0.001), and retinol metabolism as the primary deactivated pathway (*p* < 0.001). Relevant to actin cytoskeleton regulation, organelle transfer via tunneling nanotubes with MSC treatment has been previously described (Islam et al., 2012). Since actin cytoskeleton proteins are known to directly interact with mitochondria for migratory purposes (Stephen et al., 2015), elevated activity of this pathway may be related to the movement of healthy MSC donor mitochondria to sites of mitochondrial impairment and metabolic stress (Anesti and Scorrano, 2006).

### Liver fatty acid profile is differentially affected by MSC therapy

**Figure 7:**
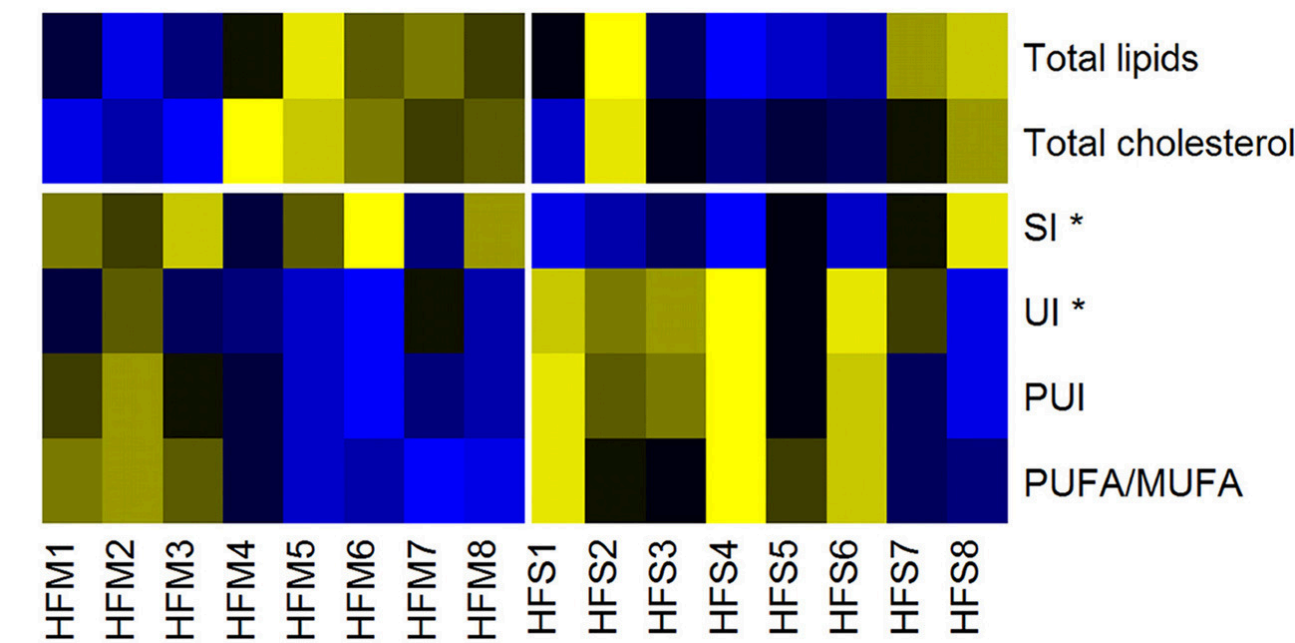
Heat map of lipid liver metabolites/indices, measured by nuclear magnetic resonance. Yellow, High concentrations; Black, Medium concentrations; Blue, Low concentrations. SI, Saturation Index; UI, Unsaturation Index; PUI, Polyunsaturation Index; PUFA/MUFA, Polyunsaturated Fatty Acids/Monounsaturated Fatty Acids. High-fat fed animals were separated into two groups for analysis: MSC treated (HFM) and saline control (HFS) ^*^denotes statistical significance at *p* < 0.05.

To understand how MSC induced alterations in mitochondrial morphology and function may be impacting metabolism, tissue specific metabolomics analyses were performed by proton nuclear magnetic resonance spectroscopy. Both aqueous and non-aqueous fractions were examined. No alterations between HFM or HFS were observed for any TCA intermediates or other aqueous metabolites (*p* > 0.05). However, an increase in lipid saturation and decrease in lipid unsaturation were identified in the HFM group (*p* < 0.05), while total lipid content remained constant (Figure 7).

## Discussion

Over the past decade the number of clinical trials involving MSCs has steadily increased, resulting in MSCs being the most commonly used cell type in tissue engineering and regenerative medicine (Wei et al., 2013). However, the nature and function of MSCs remains unclear, often impeding the effectiveness in treating various diseases (Squillaro et al., 2016). As a complex and multi-factorial disease phenotype, altered mitochondrial morphology and respiration is a feature of many chronic diseases, including those involving the heart (Rosca and Hoppel, 2013), skeletal muscle (Báez et al., 2015), liver (Lane et al., 2016), and brain (Navarro and Boveris, 2010). We therefore set out to examine both *in vitro* cell culture and *in vivo* animal experimental models to study the therapeutic impact of MSC treatment on mitochondrial function and dynamics. Major findings of this study are as follows: (1) MSC contact co-culture rescues aberrant mitochondrial morphology in skin fibroblasts from patients diagnosed with pathogenic mtDNA mutations; (2) in the context of a high fat diet, MSCs successfully xenograft into mouse liver; (3) MSC therapy increases both metabolic capacity and ROS production of the host liver; and (4) MSC therapy results in widespread host gene shifting as well as metabolomic alterations to lipid saturation within the liver.

As highly dynamic organelles, mitochondria undergo constant flux between fission and fusion morphology. Collectively termed mitochondrial dynamics, both fission and fusion structures have been tightly linked to mitochondrial function and metabolism (Liesa et al., 2009). Excessive fission has been associated with decreased membrane potential, ATP synthesis, oxygen consumption, and increased proton leak, while excessive fusion induces the opposite phenotype (elevated membrane potential, ATP synthesis, oxygen consumption, and decreased proton leak) (Sebastián et al., 2017). Known to confer metabolic benefits through the transfer of healthy mitochondria to damaged tissues (Ahmad et al., 2014), the role of MSCs to impact mitochondrial dynamics of the host mitochondrial pool is unknown. To this end, we performed a contact co-culture with MSCs comparing skin fibroblasts from healthy controls to mitochondrial disease patients (Figures 1, 2). The baseline fragmentation phenotype in patient fibroblasts was rescued by co-culture with MSCs, shifting the mitochondrial morphology to resemble control cells. Associated with improved metabolic output, shifting to an elongation morphology suggests an improvement to existing mitochondrial dysfunction. Live cell imaging was also performed and captured the presence of labeled mitochondrial components transiting from MSCs to fibroblasts (Video S1). The previously identified phenomenon of mitochondrial transfer may provide evidence of how MSCs alter fibroblast mitochondrial dynamics (Islam et al., 2012). On the other hand, mitochondrial function (i.e., OXPHOS) was not measured directly since contact co-culturing of MSCs and fibroblasts resulted in cell populations being difficult to separate, although future research should investigate the possibility of paracrine actions between neighboring cells. Finally, patient cells also exhibited a decrease in mtDNA aggregation after MSC co-culture, a trait linked to mtDNA stress (Alán et al., 2016). To further evaluate the capacity of MSCs to impact host mitochondrial function, an *in vivo* animal model was studied.

Previously, our lab demonstrated that allogeneic administration of mouse-derived MSCs migrate to multiple tissues following administration, are promoted to engraft in animals consuming a HF, and mitigate hepatic oxidative stress in HF animals (Nyamandi et al., 2013). Inviting further exploration, we examined whether human-derived MSC administration would yield similar beneficial effects to the liver of HF animals. In the present study, we performed sequence-specific PCR to detect human transcripts from donor MSCs (Figure 4). Compared to our previously published work using flow cytometry—to quantitate the number of MSCs as they localize to various tissues (Nyamandi et al., 2013)—our present technique qualitatively identified MSCs by sequence-specific PCR, exclusive to the liver of HFM animals. These results confirmed that the transplanted MSCs successfully migrated to the liver and may therefore provide metabolic improvements to surrounding host tissue. Although this method enables detection of DNA variations between species, visualization via similar immunofluorescent staining as performed for our *in vitro* cell culture experiments would have been optimal. As mentioned previously, the liver was chosen because of its inherent ability to attract stem cells caused by lipid accumulation, its high concentration of mitochondria, and the presentation of metabolic inflammation following prolonged high-fat feeding (Ren et al., 2012; Zhao et al., 2015).

As a highly metabolically active tissue the liver contains a high concentration of mitochondria. The prolonged insult of metabolic inflammation induced by the HF phenotype has been shown to diminish healthy mitochondrial populations, resulting in decreased functional capacity of oxidative phosphorylation (OXPHOS) machinery (Satapati et al., 2015). Known to confer some of their benefits through the physical transfer of healthy donor mitochondria to the impaired host (Torralba et al., 2016), we examined the impact of MSC therapy to alter host mitochondrial function in the liver. As the most commonly dysfunctional enzyme complex of the electron transport system as observed in humans, complex I activity was examined using malate and glutamate as substrates (Mimaki et al., 2012). Animals treated with MSCs exhibited a greater maximal oxygen consumption rate (complex I) as well as elevated citrate synthase enzyme activity (Table 2). Collectively these results suggest that the improvements to metabolic function with MSCs may be due to increased mitochondria present within the host liver tissue. However, there is controversy since some research has suggested that while MSCs can rescue host mitochondrial function by transferring healthy mitochondria, cells with pathogenic mtDNA mutations do not undergo rescue (Cho et al., 2012). Contrarily, our results using an *in vitro* model of mitochondrial dysfunction show that MSC co-culture can alter mitochondrial morphology of cells from patients diagnosed with pathogenic mtDNA mutations (Table 1).

Given that metabolic inflammation has been associated with an accumulation of increased mtDNA damage and impaired oxidative capacity (Yuzefovych et al., 2013; Arruda et al., 2014), the increase in citrate synthase activity following prolonged HF consumption indicates a potential increase to the viable mitochondrial population. As a by-product of healthy OXPHOS chemistry, ROS can positively impact surrounding tissues through improved cell signaling and transcriptional regulation, or negatively by oxidizing proteins and damaging DNA (Zorov et al., 2014). Since complexes I and III are the primary sites of ROS production (Brand, 2010), the collective increases in mitochondrial abundance and complex I mediated respiration in MSC treated animals may have resulted in elevated ROS production (Figure 5). The produced ROS is ultimately converted to H_2_O_2_ and sequestered by the glutathione and thioredoxin-2 (Trx2) antioxidant pathways (Stanley et al., 2011). Interestingly, recent research utilizing an *in vivo* model of ischemia-reperfusion injury has indicated that MSC therapy causes a decrease in Trx2 mRNA expression and that fibroblasts cultured in MSC conditioned media produce less ROS (Motegi et al., 2017). Since the ROS measurements in these experiments were collected over 2 h, these data may provide insight to the pathway of ROS sequestration over a prolonged period. Contrarily, our measured elevations in ROS following MSC therapy were collected over minutes and were assayed using isolated mitochondria. These differences may demonstrate that although the total ROS generated are tempered by MSC therapy, snapshots in time using shorter experimental durations may capture some of the natural variability of ROS production under stressful respiratory activity (Aon et al., 2012). We next used a gene microarray to study global gene expression patterns between groups.

The most differentially expressed functional categories yielded several commonly increased functions including cell homing, cell migration, and cell recruitment (Figure 6, Table 4). Although counterintuitive, as MSCs are known to possess immunomodulatory properties, research investigating the manner in which MSCs migrate and home to tissues of interest has eluded to a similar mechanism as described for leukocyte homing (De Becker and Van Riet, 2016). This is supported by the significant upregulation of two acute phase proteins produced by hepatocytes-serum amyloid A 1 (SAA1) and orosomucoid 1 (ORM1) which may act as hepatic signaling molecules following MSC therapy. Previously, SAA1 has been shown to be upregulated in bone-marrow and adipose derived MSCs and has been implicated in elevated energy-reserve and cholesterol metabolism (Liu et al., 2007). MSC lack expression of major histocompatibility complex (MHC) class II antigens (Le Blanc and Ringdén, 2005), and have been shown to inhibit B lymphocyte proliferation, chemotaxis, and differentiation (Corcione et al., 2005). This may enable MSCs to escape immune recognition by B lymphocytes while simultaneously promoting increased expression of genes responsible for cell homing, migration, and recruitment capacity (Chen et al., 2008). Therefore, it is not surprising to identify that both hematopoietic and non-hematopoietic cell types are characterized by similar functional increases in cell homing and migration following MSC therapy. Further investigating the response to MSCs, we identified organ inflammation, quantity of leukocytes, and activation of B lymphocytes as the primary decreased functions (Figure 6). The prolactin receptor (PRLR) enables the action of prolactin on several sites throughout the body, namely the mammary glands, brain, and prostate (Sackmann-Sala et al., 2015). PRLR has, however, been identified on MSCs and other hematopoietic cell lineages and demonstrated capacity for MSC differentiation and B lymphocyte proliferation (Sackmann-Sala et al., 2015). Downregulation of PRLR may therefore enable MSCs to migrate to their tissue of interest prior to differentiating. Previously shown to reduce systemic organ inflammation (Mei et al., 2010), suppress B lymphocyte differentiation (Corcione et al., 2005), and inhibit the formation of mixed lymphocyte cultures (Le Blanc et al., 2003), our data suggest that MSCs recapitulate this phenotype in a model of metabolic inflammation.

Further examination of the processes involved in cell migration with MSC treatment pinpointed actin cytoskeleton regulation as an upregulated key pathway. Actin cytoskeleton regulation has been implicated in modulating stem cell signaling and responsiveness (Müller et al., 2013). Through modifications of extracellular matrix composition, mechanical and molecular contact with stem cells can influence viability, and rate of engraftment (Battista et al., 2005). Although the exact role of actin cytoskeleton involvement was not examined in the present study, upregulation of this key pathway exclusive to HFM animals may demonstrate a host response to promote MSC viability and an elevated capacity for mitochondrial transfer via tunneling nanotubes (Islam et al., 2012). Furthermore, mitochondria are known to interact with cytoskeletal adaptor proteins (namely Miro1) for appropriate cellular distribution and regulation of mitochondrial morphology (Anesti and Scorrano, 2006; Ahmad et al., 2014). An upregulation of this pathway may therefore be in response to increases in mitochondrial abundance, as indicated by elevated citrate synthase activity and oxygen consumption rates following MSC therapy.

To provide a more comprehensive examination of the impact that MSC therapy had on the liver, we also explored metabolomic profiles. Metabolomics analysis revealed a significant increase in lipid saturation and a significant decrease in lipid unsaturation in the MSC treated group, while total lipid content remained constant (Figure 7, Table S2). This indicates a switch from polyunsaturated and monounsaturated fatty acids (PUFAs and MUFAs) to saturated fatty acids in animals treated with MSCs. Such a decrease in liver lipid unsaturation may be indicative of increased ROS, as the removal of oxidation-induced lipid peroxides by constitutive recycling of membrane phospholipids has been shown to reduce intracellular PUFA levels (Girón-Calle et al., 1997). Previous research investigating the role of PUFAs and MUFAs on oxidative metabolism using hepatic-derived HepG2 cells has shown that PUFAs enhance expression of ROS eliminating enzymes while MUFAs promote fatty acid oxidation and synthesis (Kohjima et al., 2009). These results indicate that variations in fatty acid composition may dictate ROS enzyme expression, ROS generation, and ROS sequestration. This coincides with our metabolic data in which we identified an increased level of ROS that was matched with increased mitochondrial abundance in the liver of MSC treated animals. Furthermore, the decreased PUFAs found in MSC treated livers may explain our elevated levels of ROS since the Trx2 mediated antioxidant pathway has been shown to be downregulated in MSC treated animals whereas elevated MUFAs promote fatty acid oxidation and may generate excess ROS (Kohjima et al., 2009; Aon et al., 2012). In contrast, the water-soluble liver metabolites showed no significant difference between groups.

As a complex biological therapy, MSCs require exploration to identify the mechanisms of beneficial action. Our data indicate that MSC therapy rescues impaired mitochondrial morphology using an *in vitro* cell culture model, enhances host metabolic capacity, and induces widespread host gene shifting in an *in vivo* animal-model of metabolic inflammation. These data may provide insight into how MSCs exert some of their therapeutic impacts and help inform future clinical targets for improving host mitochondrial function.

## Author contributions

JS, DH, TS, and AK designed and developed the research. CN, RS, MK, and JS conducted experiments, collected and analyzed data. CN and JS wrote the manuscript and all authors revised the manuscript critically for important intellectual content prior to final approval. All authors approved the final version of this manuscript, agree to be accountable for all aspects of the work in ensuring that questions related to the accuracy or integrity of any part of the work are appropriately investigated and resolved, and all persons designated as authors qualify for authorship, and all those who qualify for authorship are listed.

### Conflict of interest statement

The authors declare that the research was conducted in the absence of any commercial or financial relationships that could be construed as a potential conflict of interest.

## Supplementary material

Table S1 Primers for gene microarray validation using qRT-PCR and species specific qualitative PCR.

Table S2 P-values of liver metabolic indicies.

Figure S1 Mitochondrial morphology and mtDNA scoring classifications using a Zeiss LSM 700 confocal microscope and a 40x oil objective. Both fixed individual and live co -cultured skin fibroblast cells were manually classified using the following standardized images: A) 1 of 5 mitochondrial morphology categories and B) 1 of2 categories for mtDNA structure. Mitochondrial morphology for skin fibroblasts collected using immunofluorescence against TOMM20.

Figure S2 Typical trace of oxygen consumption following isolated mitochondria preparation using glutamate + malate as substrate, in liver tissue of C57BL/6 mice fed a high-fat diet. The blue line represents oxygen concentration within the chamber. The red line is the negative slope of the blue line (i.e., respiratory rate), normalized to tissue mass.

Video S1 Bidirectional transfer of mitochondrial components between live fibroblasts co-cultured with MSCs. Video collected using confocal microscopy demonstrating bidirectional transfer of mitochondrial components between live fibroblasts co-cultured with MSCs. Images were collected following fluorescent labeling of fibroblasts with MitoTracker Deep Red (mitochondria, red) and PicoGreen (DNA, green). MSC's were separately fluorescently labeled with MitoTracker Red (mitochondria, teal) prior to co-culture.
