# Supplementary material for: Mesenchymal Stem Cells Shift Mitochondrial Dynamics and Enhance Oxidative Phosphorylation in Recipient Cells
Source: Front Physiol. 2018 Nov 13;9:1572. doi: 10.3389/fphys.2018.01572 (PMC6282049; doi:10.3389/fphys.2018.01572)
Supplement: Table S1 — Primers for gene microarray validation using qRT-PCR and species specific qualitative PCR. [file Table_1.DOCX]

**Table S1.** Primers for gene microarray validation using qRT-PCR and species specific qualitative PCR.

| **Gene** | **Forward Primer (5'-3')** | **Reverse Primer (5'-3')** |
| --- | --- | --- |
| LCN2 | CCAGTTCGCCATGGTATTTTTC | CACACTCACCACCCATTCAGTT |
| SAA1 | CCGTTTCTGGGGTTAATG | TTCTGCTCCCTGCTCCTG |
| LPL | AGGGCTCTGCCTGAGTTGTA | AGAAATTTCGAAGGCCTGGT |
| RETSAT | TGAATGCAAGCGAGGAAAGA | CCATGGCTTGGATGGAGATT |
| ACOT1 | CTGGCGCATGCAGGATC | CCTCGATAGGTTCTTTTCACGG |
| PRLR | AAGCCAGACCATGGATACTGGAG | AGCAGTTCTTCAGACTTGCCCTT |
| β-actin | ACGGCCAGGTCATCACTATTC | AGGAAGGCTGGAAAAGAGCC |
| PTGER2 (M) | CCTGCTGCTTATCGTGGCTG | GCCAGGAGAATGAGGTGGTC |
| PTGER2 (H) | GCTGCTTCTCATTGTCTCGG | GCCAGGAGAATGAGGTGGTC |
