# Supplementary material for: Mesenchymal Stem Cells Shift Mitochondrial Dynamics and Enhance Oxidative Phosphorylation in Recipient Cells
Source: Front Physiol. 2018 Nov 13;9:1572. doi: 10.3389/fphys.2018.01572 (PMC6282049; doi:10.3389/fphys.2018.01572)
Supplement: Table S2 — P-values of liver metabolic indicies. [file Table_2.DOCX]

**Table S2.** P-values of liver metabolic indicies.

|  | **p-value** |
| --- | --- |
| **Total lipids** | 0.793 |
| **Total cholesterol** | 0.875 |
| **SI*** | 0.041 |
| **UI*** | 0.024 |
| **PUI** | 0.104 |
| **PUFA/MUFA** | 0.128 |

SI: Saturation Index, UI: Unsaturation Index, PUI: Polyunsaturation Index, PUFA/MUFA: Polyunsaturated Fatty Acids/Monounsaturated Fatty Acids *p<0.05
