# Supplementary figures and images for: Mesenchymal Stem Cells Shift Mitochondrial Dynamics and Enhance Oxidative Phosphorylation in Recipient Cells
Source: Front Physiol. 2018 Nov 13;9:1572. doi: 10.3389/fphys.2018.01572 (PMC6282049; doi:10.3389/fphys.2018.01572)

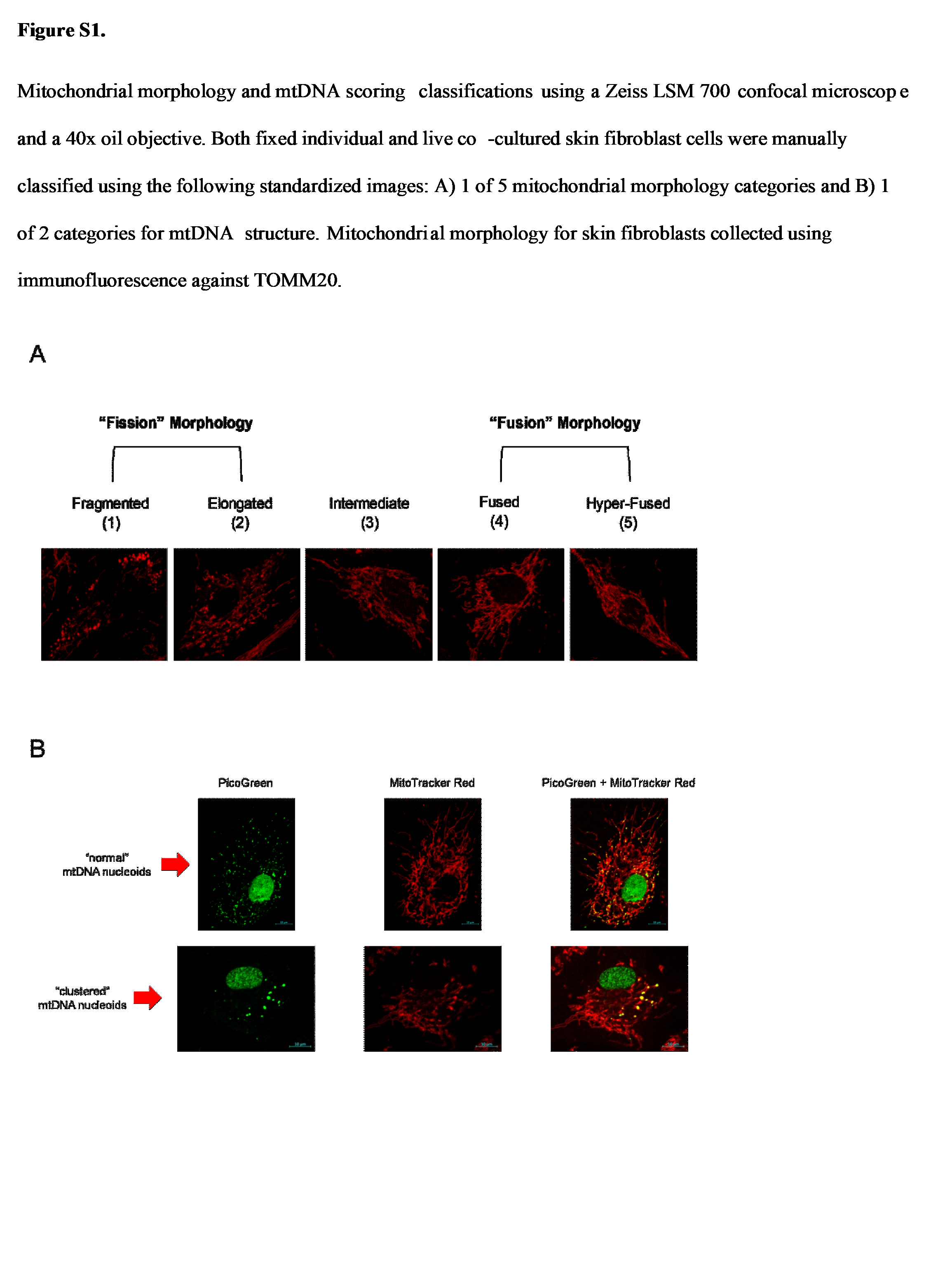

Supplement: Figure S1 — Mitochondrial morphology and mtDNA scoring classifications using a Zeiss LSM 700 confocal microscope and a 40x oil objective. Both fixed individual and live co -cultured skin fibroblast cells were manually classified using the following standardized images: A) 1 of 5 mitochondrial morphology categories and B) 1 of2 categories for mtDNA structure. Mitochondrial morphology for skin fibroblasts collected using immunofluorescence against TOMM20. [file Image_1.TIF]

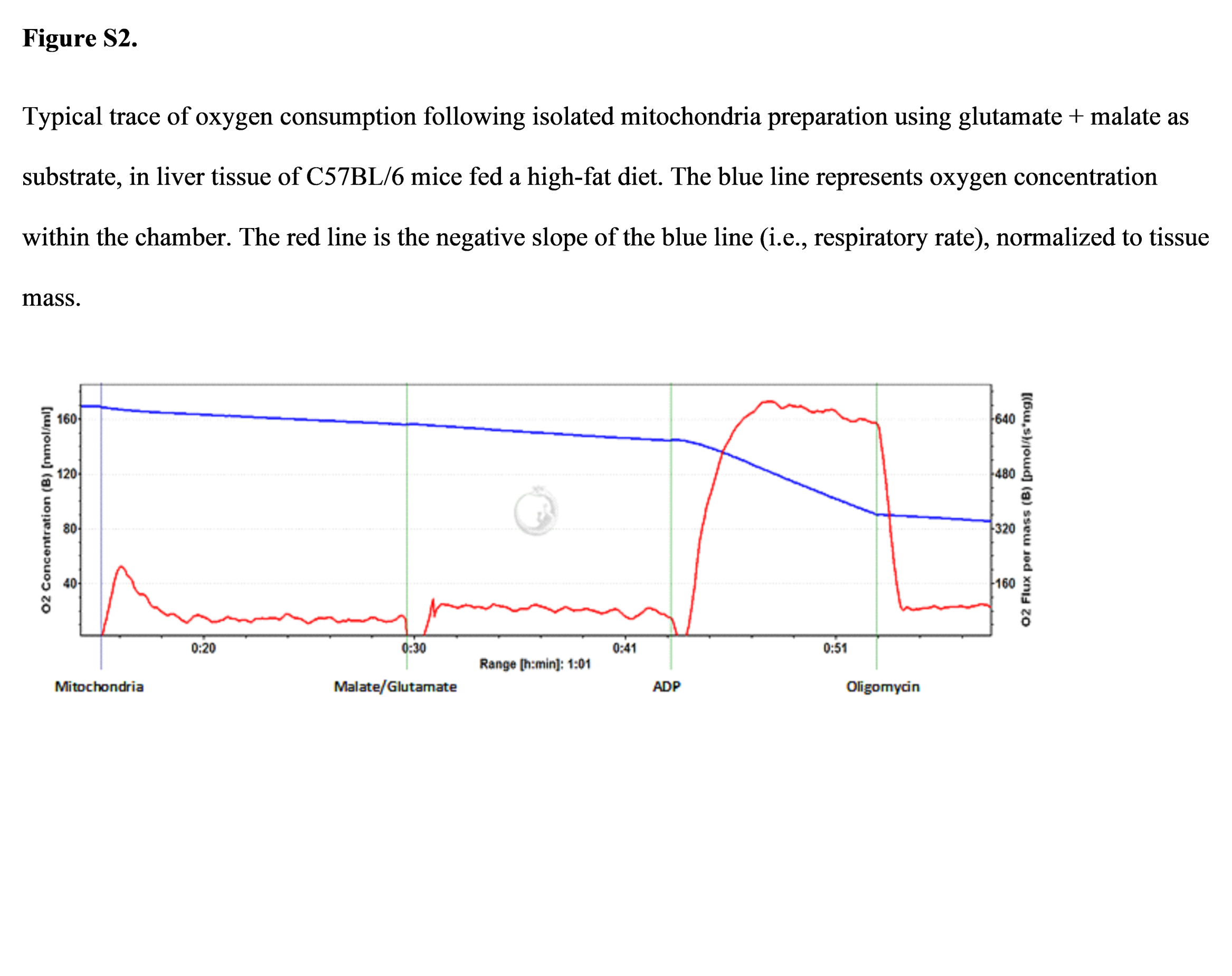

Supplement: Figure S2 — Typical trace of oxygen consumption following isolated mitochondria preparation using glutamate + malate as substrate, in liver tissue of C57BL/6 mice fed a high-fat diet. The blue line represents oxygen concentration within the chamber. The red line is the negative slope of the blue line (i.e., respiratory rate), normalized to tissue mass. [file Image_2.TIF]
